# Joint Trajectories of Death Anxiety and Experiential Avoidance After Cancer Diagnosis: A Longitudinal Study

**DOI:** 10.3390/curroncol33090546

**Published:** 2026-09-09

**Authors:** Yiguo Deng, Furong Chen, Siyu Li, Qihan Zhang, Jiaying Li, Zengjie Ye

**Affiliations:** 1 School of Nursing, Guangzhou Medical University, Guangzhou 511436, China; 2 The Nethersole School of Nursing, Faculty of Medicine, The Chinese University of Hong Kong, Hong Kong SAR, China

**Keywords:** death anxiety, experiential avoidance, dual-trajectory model

## Abstract

**Background:** Despite the observed association between death anxiety and experiential avoidance, their joint short-term trajectories in patients with newly diagnosed cancer remain unclear. This study aimed to identify joint trajectories of death anxiety and experiential avoidance during early cancer care. **Methods:** This secondary longitudinal analysis included 266 adults with newly diagnosed cancer recruited at Hunan Cancer Hospital, China, between April and September 2022. Death anxiety and experiential avoidance were assessed using the 15-item Templer Death Anxiety Scale (DAS) and the seven-item Acceptance and Action Questionnaire-II (AAQ-II), respectively, at hospital admission, discharge, and one month after discharge. This interval represented the acute adaptation phase after diagnosis and treatment initiation. Outcome-specific trajectory models informed a fully crossed dual-trajectory model. **Results:** Participants (mean age, 48.33 ± 11.18 years; 53.8% male) followed three jointly re-estimated declining DAS trajectories (low, 37.3%; moderate, 26.5%; high, 36.3%) and two declining AAQ-II trajectories (low, 70.6%; high, 29.4%). In the dual-trajectory model (entropy = 0.849), the probability of high-AAQ-II increased across low, moderate, and high-DAS trajectories (8.5%, 18.7%, and 58.6%), and 72.3% of the high-AAQ-II trajectory followed the high-DAS trajectory. A high-DAS/low-AAQ-II combination accounted for 15.0%, indicating incomplete correspondence. **Conclusions:** During early care after cancer diagnosis, high-AAQ-II membership was concentrated in higher DAS trajectories, whereas high-DAS could also accompany low-AAQ-II.

## Simple Summary

A new cancer diagnosis can bring intense fear of death. Some patients respond by trying to suppress or escape difficult thoughts, emotions, bodily sensations, or reminders of illness. We followed 266 patients from hospital admission to one month after discharge and found that death anxiety and this avoidant response generally declined. Greater avoidance was more common among patients with greater death anxiety, but some patients experienced high death anxiety without high avoidance. These findings show that different patterns of change in death anxiety were accompanied by different patterns of avoidance. This exploratory study provides a more detailed understanding of how death anxiety and avoidance change together during early cancer care and broadens evidence on patients’ psychological responses after diagnosis.

## 1. Introduction

Cancer remains a major public health burden in China, with an estimated 4.82 million new cancer cases in 2022 [1]. A cancer diagnosis is accompanied by uncertainty about treatment, prognosis, and survival [2]. This uncertainty is associated with substantial psychological distress [3]. Death anxiety is particularly relevant during the early period after cancer diagnosis. In a study of adults with primary brain tumors, 81% reported moderate or severe death anxiety, with higher levels observed among patients assessed closer to diagnosis [4]. A systematic review further showed that death anxiety was associated with greater symptom burden, anxiety, depression, fear of recurrence, and psychological distress, as well as poorer daily functioning and quality of life [5]. Therefore, death anxiety warrants attention during early cancer care.

Experiential avoidance is also relevant during early cancer care. It refers to unwillingness to remain in contact with unwanted thoughts, emotions, memories, and bodily sensations, together with efforts to control or escape these internal experiences [6]. Avoidant responses are already evident soon after cancer diagnosis [7]. The function of experiential avoidance may vary over time. A systematic review found that avoidance can serve a short-term protective function by helping patients maintain normality, hope, and distance from overwhelming emotions, whereas persistent avoidance was associated with poorer functioning and reduced engagement in life over longer periods [8]. Within the Psychological Flexibility Model, persistent experiential avoidance represents an important component of psychological inflexibility. Identifying different patterns of experiential avoidance during early cancer care may therefore help characterize differences in how patients respond to distressing internal experiences after diagnosis [9,10].

Existing evidence supports a close association between experiential avoidance and death anxiety in patients with cancer. Greater experiential avoidance has been associated with greater death anxiety [11], whereas greater experiential acceptance has been associated with lower death anxiety [12]. However, it still remains unclear how different trajectories of death anxiety and experiential avoidance develop jointly during early cancer care. Group-based dual-trajectory modeling can estimate the joint and conditional probabilities of these longitudinal patterns. This approach can characterize how different trajectories of death anxiety and experiential avoidance co-occur and develop jointly over time [13,14].

Accordingly, this study aimed to characterize the joint trajectories of death anxiety and experiential avoidance during early cancer care and to examine baseline characteristics associated with trajectory membership. These findings may support a more differentiated understanding of psychological responses during early cancer care and inform future psychosocial assessment research.

## 2. Methods

### 2.1. Study Design and Participants

This study was a secondary analysis of data from a longitudinal observational study of death anxiety among patients with newly diagnosed cancer [15]. Between April and September 2022, the parent study recruited patients by consecutive sampling from clinical departments at Hunan Cancer Hospital in China. The parent study was approved by the Medical Ethics Committee of Hunan Cancer Hospital (2022 Scientific Research Expedited Review No. 5), and all participants provided written informed consent.

Eligible patients were aged 18 years or older, had a first pathologically confirmed malignancy, were enrolled within 7 days of initiating anticancer treatment, were aware of their diagnosis, and had normal cognition (Chinese Mini-Mental State Examination score, 27–30). Patients were excluded if they had a documented psychiatric or psychological disorder, communication or reading difficulties, or a clinical condition that precluded participation. Assessments were conducted at hospital admission (T1), hospital discharge (T2), and 1 month after discharge (T3), with each assessment completed within 7 days of the scheduled time point. If a participant experienced marked emotional distress during questionnaire completion, the assessment was paused, and psychological support was provided. Of the 300 patients approached, 283 completed a valid baseline assessment and 266 completed all three assessments, constituting the complete-case analytic cohort.

### 2.2. Sample Size

The sample size calculation followed that of the parent longitudinal study and was not separately performed for the present secondary dual-trajectory analysis. The parent study reported using the formula $N=(4t_{\alpha }^{2}s^{2})/\delta^{2}$, with $t_{\alpha }=1.96$ at a two-sided significance level of 0.05, an estimated standard deviation of 3.05 derived from previous death-anxiety research, and an allowable error set at 0.25 times the standard deviation. This calculation yielded a reported minimum sample size of 245 participants. After allowing for an anticipated sampling loss of 10–15%, the planned sample size was increased to 281. Finally, 266 participants constituted the analytic cohort.

### 2.3. Measures

Death anxiety was measured at each occasion using the 15-item Templer Death Anxiety Scale (DAS) [16]. The scale covers cognitive, affective, temporal-awareness, and stress-and-suffering components. Items are scored yes (1) or no (0), with six items reverse-scored; total scores range from 0 to 15, and higher scores indicate greater death anxiety. The continuous total score was used in the trajectory models. The parent study reported a Cronbach α of 0.85.

Experiential avoidance was assessed using the seven-item Acceptance and Action Questionnaire-II (AAQ-II) [17]. Experiential avoidance refers to an unwillingness to remain in contact with unwanted thoughts, emotions, bodily sensations, or memories, together with attempts to control or escape these internal experiences. Items are rated from 1 to 7, yielding a total score of 7–49; higher scores indicate greater experiential avoidance and lower psychological flexibility. The questionnaire assesses general experiential avoidance rather than avoidance specifically related to death or cancer. Cronbach α was 0.71 in the present sample.

Baseline covariates comprised age, sex, education, marital status, residence, monthly income, cancer site, cancer stage, pain score, and Karnofsky Performance Status (KPS) [18]. Age and KPS were modeled per 10-unit increase. Education was classified as junior high school or lower, high school or vocational school, and college or higher; monthly income as <3000, 3000–4999, and ≥5000 Chinese yuan (CNY; equivalent to approximately <$417, $417–$694, and ≥$694 USD, based on an exchange rate of 1 USD ≈ 7.2 CNY); and cancer site as head/neck or intracranial, breast/gynecological or reproductive, respiratory, and digestive or urinary. Cancer stage was dichotomized as stage I–II versus stage III–IV. Reference categories and all recoding decisions are given in Table S1.

### 2.4. Statistical Analysis

Analyses were conducted using Mplus version 8.11 and R version 4.6.1. All statistical tests were two-sided. Participant characteristics were summarized using means and standard deviations, medians and interquartile ranges, or frequencies and percentages, as appropriate. Baseline characteristics were compared between participants who completed all three assessments and those lost to follow-up using Welch’s *t* tests for continuous variables and Fisher’s exact tests or Monte Carlo Pearson chi-square tests for categorical variables, as appropriate.

We used group-based trajectory modeling to identify outcome-specific DAS and AAQ-II trajectories [19,20]. Intercept and linear slope variances were fixed at zero within classes; accordingly, the estimated trajectories represent class-average patterns. One- to five-class linear solutions were estimated because only three occasions were available. Assessment occasions were coded as 0, 1, and 2. Because the intervals between assessments were unequal, time was modeled by assessment occasion; the slopes therefore represent average change per modeled occasion. To reduce the risk of local maxima, each model used 1000 random starting values, with 250 retained for final-stage optimization [21]. Model selection considered lower Akaike information criterion (AIC), Bayesian information criterion (BIC), and sample-size-adjusted BIC (aBIC); adjusted Lo–Mendell–Rubin likelihood-ratio test (aLMR-LRT) and bootstrap likelihood-ratio test (BLRT) *p* < 0.05; entropy ≥0.80; average posterior probability (AvePP) ≥0.70; class size ≥5%; convergence; replication of the optimum log-likelihood; and interpretability [22,23].

We used classification-error-adjusted multinomial logistic regression with the R3STEP procedure to evaluate baseline factors associated with DAS and AAQ-II trajectory membership [24]. All prespecified covariates were entered simultaneously without stepwise selection. Using the lowest trajectory class for each outcome as the reference group, the results are reported as relative risk ratios (RRRs) with 95% confidence intervals (CIs); a 2-sided *p* < 0.05 indicated statistical significance. These analyses were considered exploratory, and interpretation emphasized the magnitude and precision of the RRR estimates.

We used a fully crossed group-based dual-trajectory model to characterize the longitudinal co-occurrence of DAS and AAQ-II trajectories [20]. The model retained the class numbers and linear forms selected from the outcome-specific models and jointly re-estimated all parameters using 3000 random starting values and 750 final-stage optimizations [25]. Equality constraints ensured that combinations sharing the same DAS or AAQ-II class had the same corresponding trajectory parameters [26]. Joint probabilities estimated the prevalence of each trajectory combination; marginal probabilities were obtained by summing the relevant joint probabilities, and bidirectional conditional probabilities were calculated by dividing each joint probability by the corresponding marginal probability [27]. These probabilities describe longitudinal co-occurrence rather than temporal or causal relationships.

## 3. Results

### 3.1. Participants and Observed Scores

The analytic sample comprised 266 patients with newly diagnosed cancer (mean age, 48.33 ± 11.18 years; 53.8% male). Nearly half had a junior high school or lower education (47.7%), 94.0% were married, and 42.5% lived in urban areas. Monthly income was below 3000 yuan for 26.3%, 3000–4999 yuan for 35.3%, and at least 5000 yuan for 38.3%.

Cancer sites were head/neck or intracranial (25.2%), breast/gynecological/reproductive (24.4%), respiratory (25.2%) and digestive/urinary (25.2%). Stage III–IV disease was present in 22.2% of participants. Mean pain and Karnofsky Performance Status scores were 3.19 ± 1.65 and 72.03 ± 9.42, respectively. DAS scores were 7.77 ± 4.63 at T1, and AAQ-II scores were 22.57 ± 9.66 [15]. Baseline characteristics did not differ significantly between participants who completed all three assessments and those lost to follow-up (all *p* > 0.05) (Table 1).

### 3.2. Death Anxiety and Experiential Avoidance Trajectory Model Selection and Characteristics

The final DAS model comprised low (38.0%; slope = −1.37 per occasion), moderate (27.4%; slope = −1.18), and high (34.6%; slope = −0.79) declining trajectories. The three-class model was retained over the two-class model because it had a lower BIC (reduction = 38.102), significant aLMR-LRT (*p* = 0.011) and BLRT (*p* < 0.001), and high entropy (0.831). Although four- and five-class models further reduced BIC, they produced class proportions below the prespecified 5% threshold.

**Figure 1:**
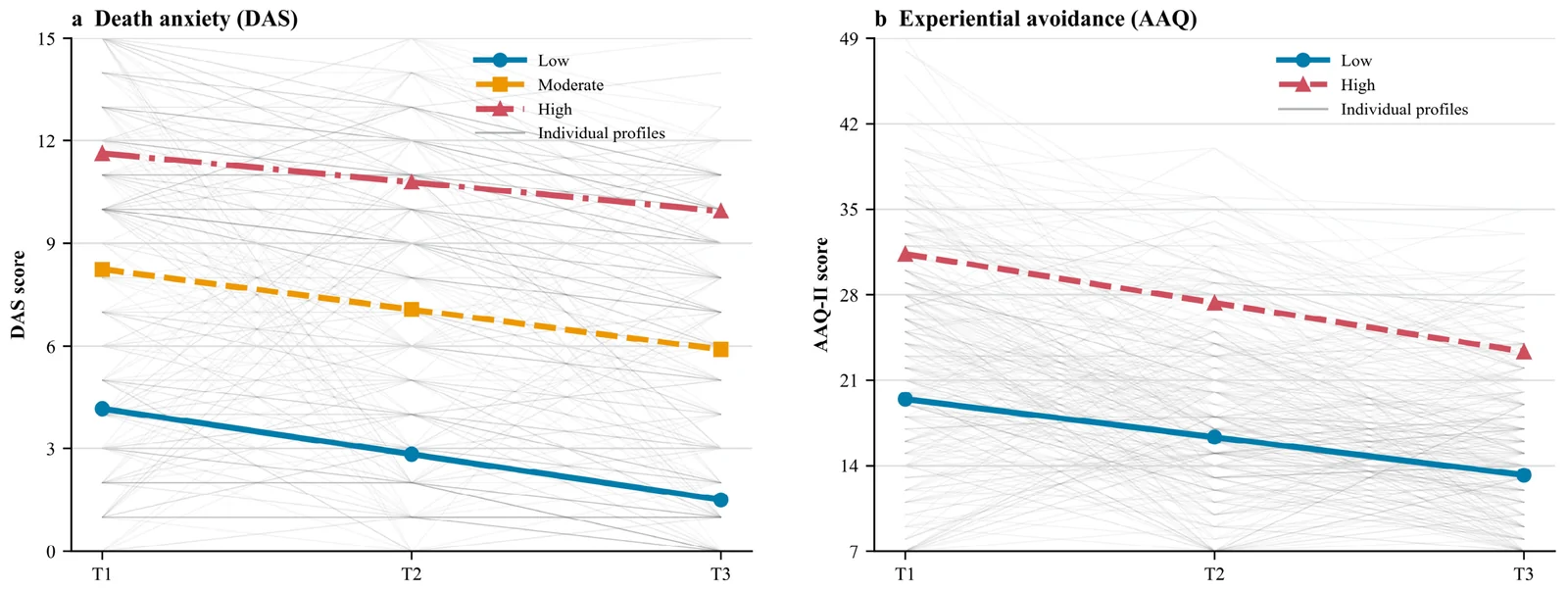
Observed individual profiles and model-estimated outcome-specific trajectories of death anxiety and experiential avoidance. Thin gray lines show observed individual scores for 266 participants, whereas colored lines show model-estimated class-average trajectories. Because within-class intercept and slope variances were fixed at zero, the colored trajectories represent class-average patterns rather than individual clinical courses. T1, hospital admission; T2, hospital discharge; T3, one month after discharge.

The final AAQ-II model comprised low-decreasing (71.6%; slope = −3.13) and high-decreasing (28.4%; slope = −3.94) trajectories. The two-class model was retained over the one-class model because it had a lower BIC (reduction = 181.810), significant aLMR-LRT (*p* < 0.001) and BLRT (*p* < 0.001), and high entropy (0.867). Higher-class models were rejected because of increased BIC, nonsignificant likelihood-ratio tests, or class proportions below 5% (Figure 1; Table 2 and Table S2).

### 3.3. Baseline Correlates of Trajectory Membership

**Figure 2:**
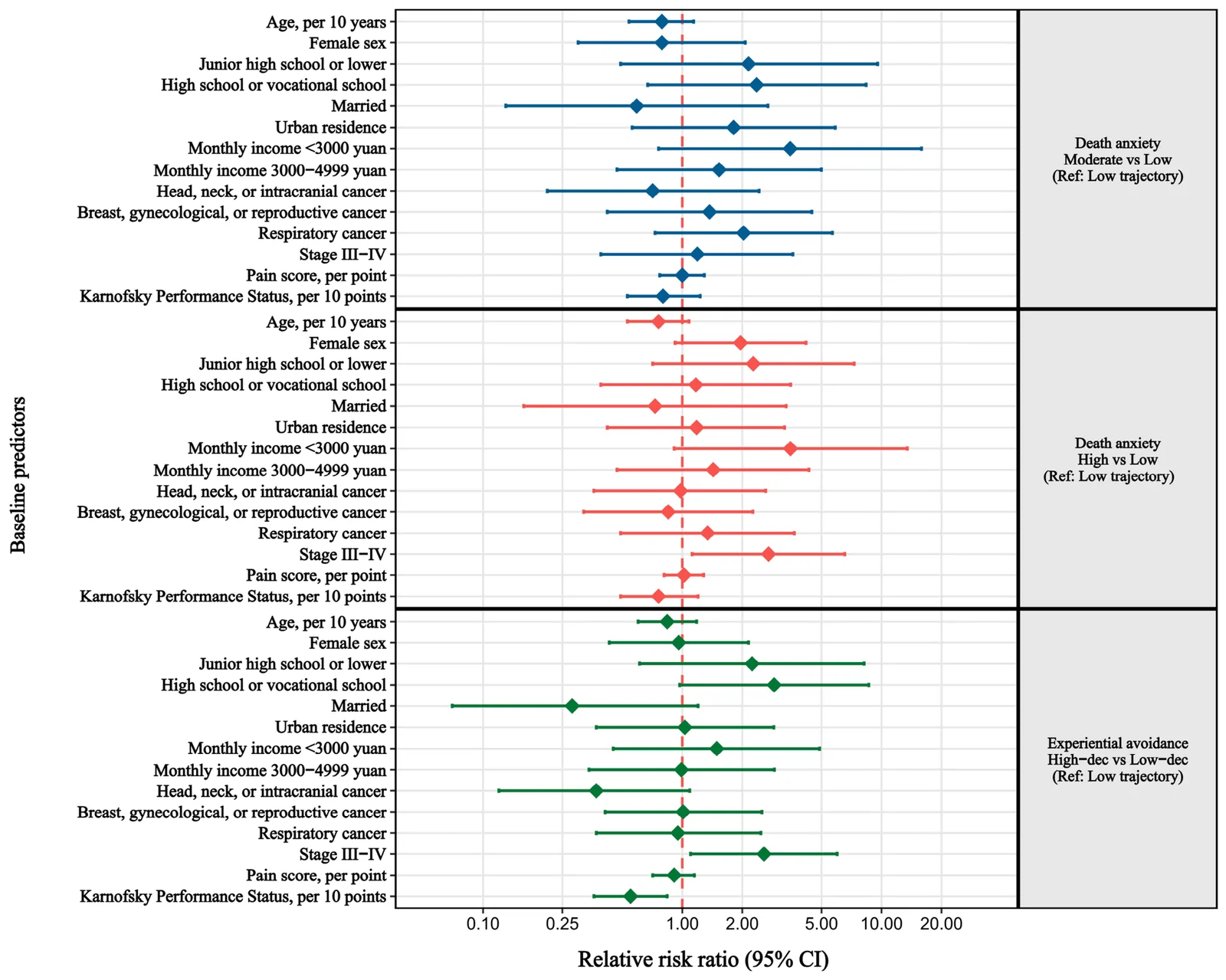
Adjusted baseline associations with death-anxiety and experiential avoidance trajectory membership. Diamonds show relative risk ratios and horizontal lines show 95% confidence intervals from classification-error-adjusted multinomial logistic regression. Each comparison uses the indicated low trajectory as the reference, and the dashed vertical line marks a relative risk ratio of 1. All prespecified covariates were entered simultaneously; estimates are exploratory.

Among the baseline covariates examined, only cancer stage and KPS reached the nominal two-sided significance level. Stage III–IV disease was associated with the high- rather than low-DAS trajectory (RRR = 2.71; 95% CI, 1.12–6.54; *p* = 0.027) and the high-decreasing rather than low-decreasing AAQ-II trajectory (RRR = 2.57; 95% CI, 1.10–5.99; *p* = 0.028). Each 10-point increase in KPS was inversely associated with high-decreasing AAQ-II trajectory membership (RRR = 0.55; 95% CI, 0.36–0.84; *p* = 0.006) (Figure 2; Table S3). These baseline-association findings should be considered exploratory.

### 3.4. Joint Trajectory Analysis

The final 3 × 2 dual-trajectory model converged normally and replicated the optimum log-likelihood across random starting values (log-likelihood = −4718.09; AIC = 9478.18; BIC = 9553.43; aBIC = 9486.85; entropy = 0.849). The jointly re-estimated marginal proportions and trajectory slopes remained substantively consistent with those obtained from the outcome-specific models. After joint re-estimation, DAS comprised low (37.3%; slope = −1.33; AvePP = 0.957), moderate (26.5%; slope = −1.17; AvePP = 0.858), and high (36.3%; slope = −0.84; AvePP = 0.953) declining trajectories. AAQ-II comprised low-decreasing (70.6%; slope = −3.10; AvePP = 0.971) and high-decreasing (29.4%; slope = −3.99; AvePP = 0.942) trajectories. Marginal AvePP values ranged from 0.858 to 0.971 (Table 3).

**Figure 3:**
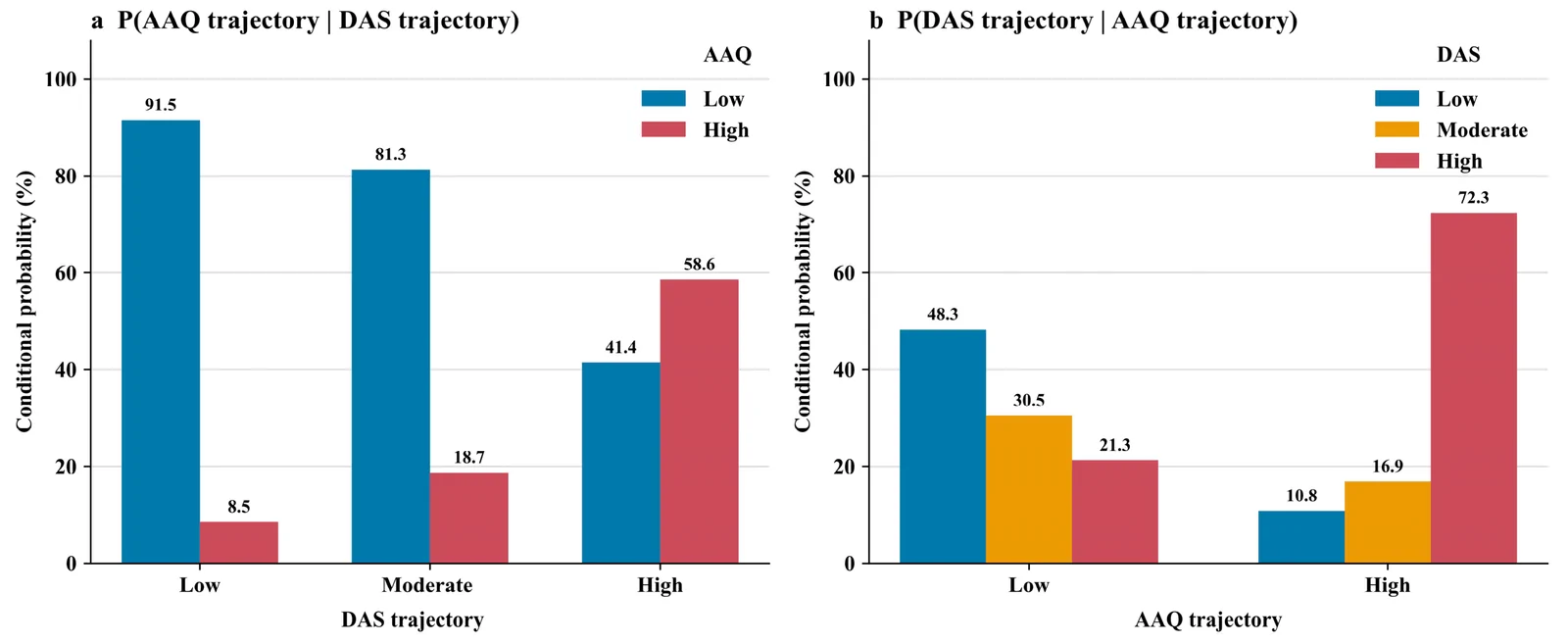
Bidirectional conditional probabilities linking death-anxiety and experiential avoidance trajectories. Panel (**a**) shows the probability of AAQ-II trajectory membership conditional on each DAS trajectory; bars within each DAS group sum to 100%. Panel (**b**) shows the probability of DAS trajectory membership conditional on each AAQ-II trajectory; bars within each AAQ-II group sum to 100%. These probabilities describe model-estimated co-occurrence, not temporal or causal relationships.

Conditional probabilities showed graded but incomplete correspondence between the DAS and AAQ-II trajectories. Conditional on DAS trajectory membership, the probability of high-decreasing AAQ-II membership increased from 8.5% in the low-DAS trajectory to 18.7% in the moderate-DAS trajectory and 58.6% in the high-DAS trajectory. Conversely, conditional on low-decreasing AAQ-II membership, the probabilities of low-, moderate-, and high-DAS membership were 48.3%, 30.5%, and 21.3%, respectively. Conditional on high-decreasing AAQ-II membership, the corresponding probabilities were 10.8%, 16.9%, and 72.3% (Figure 3; Table S4).

**Figure 4:**
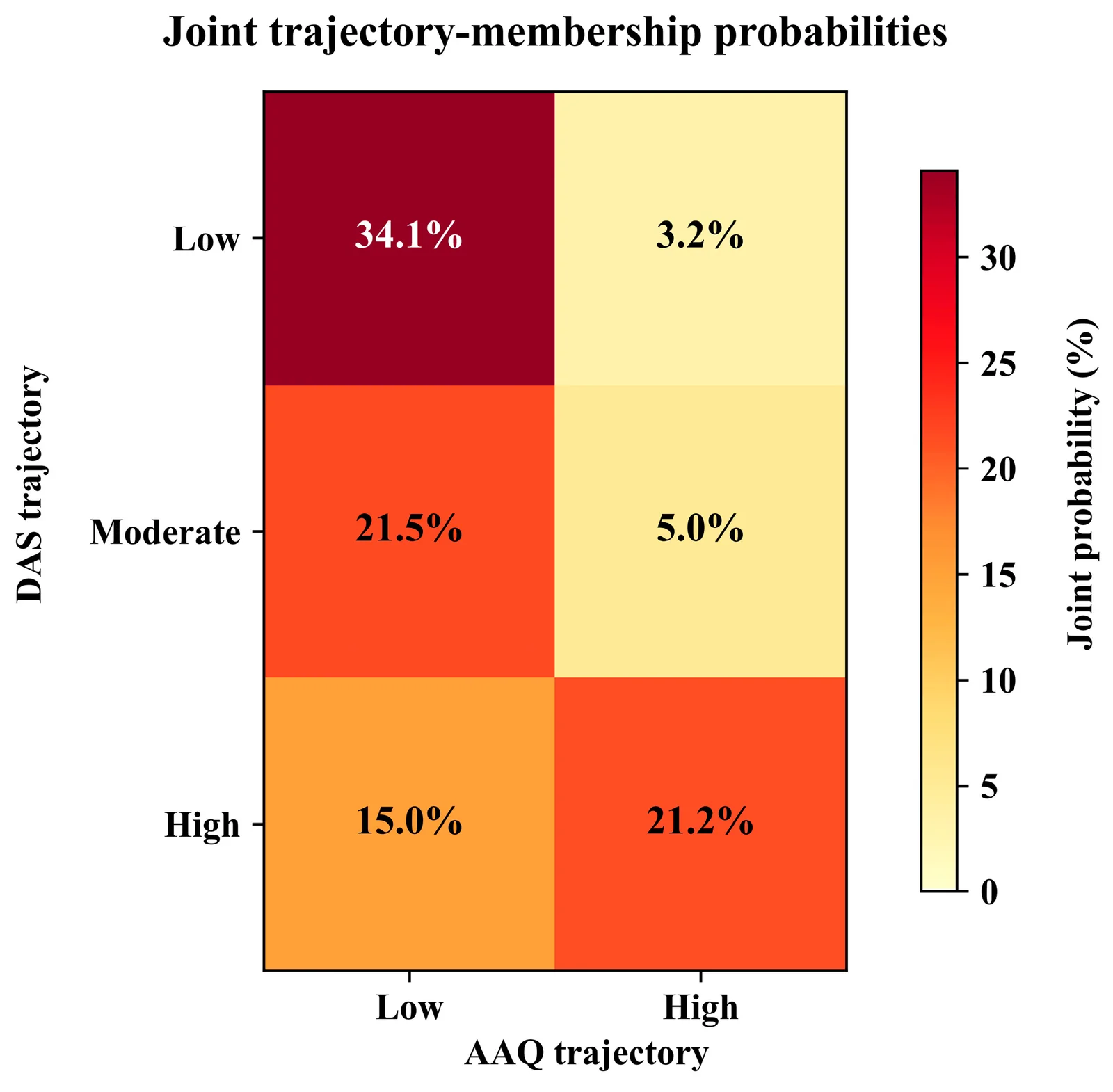
Joint probabilities of death-anxiety and experiential avoidance trajectory membership. Each cell shows the model-estimated prevalence of one DAS–AAQ-II trajectory combination; the six cells sum to 100%, apart from rounding. Joint probabilities describe co-occurrence and do not indicate temporal or causal relationships.

The largest joint trajectory combination was low-DAS–low-decreasing AAQ-II (34.1%), followed by moderate-DAS–low-decreasing AAQ-II (21.5%) and high-DAS–high-decreasing AAQ-II (21.2%). The remaining combinations were high-DAS–low-decreasing AAQ-II (15.0%), moderate-DAS–high-decreasing AAQ-II (5.0%), and low-DAS–high-decreasing AAQ-II (3.2%) (Figure 4; Table S5). The low-DAS/high-AAQ-II cell was small and was therefore interpreted as exploratory. All joint and conditional probabilities represent model-estimated co-occurrence rather than deterministic assignment or causal relationships.

## 4. Discussion

### 4.1. Summary of Findings

This longitudinal analysis identified three jointly re-estimated declining DAS trajectories and two declining AAQ-II trajectories during the acute adaptation phase after cancer diagnosis and treatment initiation. Although all trajectories declined from admission to one month after discharge, differences between trajectory levels persisted. The probability of high-AAQ-II increased across the low-, moderate-, and high-DAS trajectories, and most patients in the high-AAQ-II trajectory also followed the high-DAS trajectory. Correspondence was incomplete because high-DAS could also accompany low-AAQ-II. Stage III–IV disease was associated with the high trajectory of each outcome, whereas better physical performance was associated with a lower relative risk of high-AAQ-II membership.

### 4.2. Interpretation and Comparison

#### 4.2.1. Trajectories and Predictors of Death Anxiety

The three DAS trajectories remained separated while declining during early cancer care. Similar early reductions in cancer-related distress have been observed during treatment, although longitudinal studies also show that distress can change again with treatment transitions and emerging symptoms [28,29]. The present decline should therefore be interpreted within the specific window from admission to one month after discharge, not as evidence of sustained psychological recovery. Stage III–IV disease was associated with high- rather than low-DAS membership. This finding is consistent with evidence linking death anxiety to perceived mortality threat, symptom burden, and prognosis-related concerns [5,12]. However, stage was measured only at baseline; the result identifies a possible clinical context for closer attention.

#### 4.2.2. Trajectories and Predictors of Experiential Avoidance

The low and high-AAQ-II trajectories remained separated while declining, with the high trajectory showing a steeper decline. Because follow-up covered only the acute adaptation period, this separation cannot determine whether either pattern was transient, normative, or maladaptive. Declining AAQ-II scores indicate less measured experiential avoidance but do not establish durable psychological flexibility or adaptive functioning [8]. Better physical performance was associated with a lower likelihood of high-AAQ-II membership, but this exploratory association should not be interpreted as a determinant of avoidance.

#### 4.2.3. Dual Trajectory of Death Anxiety and Experiential Avoidance

During the early period after cancer diagnosis and treatment initiation, all jointly re-estimated DAS and AAQ-II trajectories declined. Similar short-term reductions in cancer-related distress have been reported during treatment [28,29]. Avoidance may also provide temporary distance from overwhelming emotions during cancer care [8]. Within the Psychological Flexibility Model, the concurrent declines are compatible with, but do not establish, a reduction in rigid control of difficult internal experiences as early care progresses [6,10]. Despite their shared downward direction, high-AAQ-II membership remained concentrated in higher DAS trajectories, consistent with cancer evidence linking experiential avoidance and death anxiety [12,30]. The short follow-up does not establish sustained recovery or causal coupling, and AAQ-II scores may partly reflect general negative affect [31].

At the same time, a model-estimated group followed a high-DAS/low-AAQ-II pattern, indicating that comparatively high death anxiety can occur without high measured experiential avoidance. This qualifies studies that mainly reported average associations between acceptance or experiential avoidance and death anxiety [11,30]. Death anxiety should therefore be assessed directly rather than inferred from avoidance alone. Because this pattern was model-estimated and lacks a clinical threshold, it remains exploratory and should not guide diagnosis or treatment selection. Low-AAQ-II scores also do not establish acceptance or resilience [9,31].

Clinically, death-related distress and responses to it may need to be considered separately. Strong avoidance-related responses may warrant inquiry about death anxiety, whereas high death anxiety alone should not be assumed to indicate avoidance. Repeated assessment during early care may help identify persistent or worsening distress. Stage III–IV disease and poorer physical performance may indicate contexts for closer attention, but neither these factors nor trajectory membership constitutes a screening or diagnostic criterion. Further psychosocial assessment may be appropriate when distress interferes with functioning, communication, treatment engagement, or safety [32,33].

#### 4.2.4. Limitations and Future Direction

Several limitations warrant caution. Three unequally spaced assessments covered only admission to one month after discharge and were modeled by occasion; without elapsed-time sensitivity analysis, the linear, fixed-variance specification may overstate early decline, miss nonlinear change, and obscure within-class heterogeneity. The parent study was not powered for dual-trajectory analysis, and the small joint cell, multiple covariates, wide confidence intervals, and absence of multiplicity adjustment or penalization make the joint-cell and correlate findings exploratory. The small number of participants lost to follow-up limited the precision of the attrition comparison, and complete-case analysis without a missing-data sensitivity analysis may still introduce selection bias, while the single-center sample and eligibility criteria limit generalizability. Self-reported measures, possible negative-affect contamination of the AAQ-II, absent clinical thresholds or diagnostic assessments, and exclusion of patients with documented psychological disorders preclude distinguishing expected adjustment from clinically significant responses. Baseline-only covariates omitted time-varying clinical and psychosocial factors, and joint probabilities indicate co-occurrence rather than causality. Larger multicenter studies with longer follow-up and prespecified sensitivity analyses are needed.

## 5. Conclusions

During early care after cancer diagnosis, we found that death anxiety and this avoidant response generally declined. Greater avoidance was more common among patients with greater death anxiety, but some patients experienced high death anxiety without high avoidance. These findings show that different patterns of change in death anxiety were accompanied by different patterns of avoidance. This exploratory study provides a more detailed understanding of how death anxiety and avoidance change together during early cancer care and broadens evidence on patients’ psychological responses after diagnosis.

## Figures and Tables

**Table 1:** Baseline characteristics and repeated outcome scores.

Characteristic	Total Group (*n* = 266)	Loss-to-Follow-Up Group (*n* = 17)	*p*
**Age, years**	48.33 (11.18)	51.65 (11.84)	0.277
**Sex**			1.000
Male	143 (53.8%)	9 (52.9%)	
Female	123 (46.2%)	8 (47.1%)	
**Education**			0.791
Junior high school or lower	127 (47.7%)	9 (52.9%)	
High school or vocational school	99 (37.2%)	5 (29.4%)	
College or higher	40 (15.0%)	3 (17.6%)	
**Marital status**			0.096
Unmarried	16 (6.0%)	3 (17.6%)	
Married	250 (94.0%)	14 (82.4%)	
**Residence**			0.137
Rural	153 (57.5%)	13 (76.5%)	
Urban	113 (42.5%)	4 (23.5%)	
**Monthly income, CNY**			0.203
<3000	70 (26.3%)	7 (41.2%)	
3000–4999	94 (35.3%)	7 (41.2%)	
≥5000	102 (38.3%)	3 (17.6%)	
**Cancer site**			0.220
Head/neck or intracranial	67 (25.2%)	3 (17.6%)	
Breast, gynecological or reproductive	65 (24.4%)	4 (23.5%)	
Respiratory	67 (25.2%)	2 (11.8%)	
Digestive or urinary	67 (25.2%)	8 (47.1%)	
**Cancer stage**			1.000
Stage I–II	207 (77.8%)	13 (76.5%)	
Stage III–IV	59 (22.2%)	4 (23.5%)	
**Pain score**	3.19 (1.65)	2.65 (0.79)	0.058
**KPS score**	72.03 (9.42)	71.18 (12.19)	0.780
**DAS score, T1**	7.77 (4.63)	8.00 (4.54)	0.842
**AAQ-II score, T1**	22.57 (9.66)	21.88 (10.10)	0.788

Note. Values are mean (standard deviation) or *n* (%). AAQ-II, Acceptance and Action Questionnaire-II; DAS, Death Anxiety Scale; KPS, Karnofsky Performance Status.

**Table 2:** Fit indices for one- to five-class outcome-specific trajectory models.

Outcome	Classes	AIC	BIC	aBIC	Entropy	LMR-LRT *p*	BLRT *p*	Class Probabilities (%)
Death anxiety	1	4605.757	4623.674	4607.821	—	—	—	100.0
Death anxiety	2	4252.787	4281.455	4256.091	0.874	<0.001	<0.001	53.0/47.0
Death anxiety	3	4203.935	4243.353	4208.477	0.831	0.011	<0.001	27.4/38.0/34.6
Death anxiety	4	4183.323	4233.492	4189.104	0.857	0.041	<0.001	33.8/26.7/35.0/4.5
Death anxiety	5	4149.325	4210.245	4156.345	0.898	0.016	—	5.6/34.6/4.1/33.1/22.6
Experiential avoidance	1	5511.883	5529.801	5513.948	—	—	—	100.0
Experiential avoidance	2	5319.323	5347.991	5322.627	0.867	<0.001	<0.001	28.4/71.6
Experiential avoidance	3	5321.783	5361.201	5326.325	0.895	0.055	0.545	27.9/70.8/1.3
Experiential avoidance	4	5320.464	5370.633	5326.245	0.764	0.452	—	35.9/20.7/7.2/36.3
Experiential avoidance	5	5319.001	5379.920	5326.021	0.802	0.005	—	19.3/1.4/7.1/35.4/36.8

Note. Selected solutions were the three-class model for DAS and the two-class model for AAQ-II. AIC, Akaike information criterion; aBIC, sample-size-adjusted Bayesian information criterion; aLMR-LRT, adjusted Lo–Mendell–Rubin likelihood-ratio test; BLRT, bootstrap likelihood-ratio test. A dash indicates that the statistic was not estimated. Class probabilities are ordered by Mplus class number.

**Table 3:** Jointly estimated marginal trajectory parameters and classification quality.

Outcome	Trajectory	%	Intercept	s.e.	Slope	s.e.	AvePP
Death anxiety	Low	37.3	4.162	0.427	−1.330	0.224	0.957
Death anxiety	Moderate	26.5	8.244	0.432	−1.170	0.294	0.858
Death anxiety	High	36.3	11.632	0.340	−0.842	0.186	0.953
Experiential avoidance	Low	70.6	19.429	0.490	−3.103	0.300	0.971
Experiential avoidance	High	29.4	31.341	0.828	−3.992	0.521	0.942

Note. Percentages are model-estimated marginal probabilities from the final fully crossed dual-trajectory model. Intercepts represent estimated scores at hospital admission, and slopes represent mean linear change per modeled assessment occasion. AvePP, average posterior probability; s.e., standard error. Percentages may not total 100% because of rounding.

## Data Availability

The data presented in this study are available on request from the corresponding author. The data are not publicly available due to privacy and ethical restrictions.

## Supplementary Materials

The following supporting information can be downloaded at: https://www.mdpi.com/article/10.3390/curroncol33090546/s1, Table S1: Coding of baseline covariates; Table S2: Characteristics of the final trajectory models for death anxiety and experiential avoidance; Table S3: Baseline covariates of death anxiety and experiential avoidance trajectory membership; Table S4: Conditional trajectory probabilities; Table S5: Joint probabilities of death anxiety and experiential avoidance trajectory membership.

## Abbreviations

AAQ-II	Acceptance and Action Questionnaire-II
AIC	Akaike information criterion
aBIC	sample-size-adjusted Bayesian information criterion
aLMR-LRT	adjusted Lo–Mendell–Rubin likelihood-ratio test
AvePP	average posterior probability
BIC	Bayesian information criterion
BLRT	bootstrap likelihood-ratio test
CI	confidence interval
CNY	Chinese yuan
DAS	Death Anxiety Scale
KPS	Karnofsky Performance Status
RRR	relative risk ratio
SD	standard deviation
T1	hospital admission
T2	hospital discharge
T3	one month after discharge
USD	United States dollar
