# Supplementary material for: Joint Trajectories of Death Anxiety and Experiential Avoidance After Cancer Diagnosis: A Longitudinal Study
Source: Curr Oncol. 2026 Sep 9;33(9):546. doi: 10.3390/curroncol33090546 (PMC13605088; doi:10.3390/curroncol33090546)
Supplement: Supplementary file 1 [file curroncol-33-00546-s001.zip › curroncol-4501714-supplementary.pdf]

Table S1. Coding of baseline covariates

Covariate	Reference category or unit
Age, per 10 years	Per 10 years
Female sex	Male
Junior high school or lower	College or higher
High school or vocational school	College or higher
Married	Not married
Urban residence	Rural
Monthly income <3,000 CNY	≥5,000 CNY
Monthly income 3,000-4,999 CNY	≥5,000 CNY
Head/neck or intracranial cancer	Digestive or urinary cancer
Breast, gynecological or reproductive cancer	Digestive or urinary cancer
Respiratory cancer	Digestive or urinary cancer
Stage III-IV cancer	Stage I-II
Pain score, per point	Per 1 point
Karnofsky score, per 10 points	Per 10 points

Note. Education, income and cancer site were represented by indicator variables. Age and Karnofsky Performance Status were scaled per 10 units. CNY = Chinese Yuan. At the time of the study, 1 USD was equivalent to approximately 7.2 CNY.

Table S2. Characteristics of the final trajectory models for death anxiety and experiential avoidance

Outcome	Trajectory	Model-estimated probability (%)	Intercept	Linear slope	AvePP
DAS	Low declining	38.0	4.24	−1.37	0.962
DAS	Moderate declining	27.4	8.42	−1.18	0.861
DAS	High declining	34.6	11.63	−0.79	0.937
AAQ	Low decreasing	71.6	19.54	−3.13	0.971
AAQ	High decreasing	28.4	31.48	−3.94	0.956

Note. DAS, Death Anxiety Scale; AAQ, Acceptance and Action Questionnaire-II; AvePP, average posterior probability.

Table S3. Baseline covariates of death-anxiety and experiential avoidance trajectory membership

Predictor	Moderate vs Low death anxiety RRR (95% CI)	P	High vs Low death anxiety RRR (95% CI)	P	High vs Low AAQ RRR (95% CI)	P
**Demographic Characteristics**						
Age, per 10 years	0.79 (0.54–1.14)	0.202	0.76 (0.53–1.08)	0.127	0.84 (0.60–1.18)	0.312
**Sex**						
Male	1.00 (Reference)	—	1.00 (Reference)	—	1.00 (Reference)	—
Female	0.79 (0.30–2.07)	0.638	1.96 (0.92–4.18)	0.081	0.96 (0.43–2.15)	0.93
**Marital status**						
Unmarried	1.00 (Reference)	—	1.00 (Reference)	—	1.00 (Reference)	—
Married	0.59 (0.13–2.69)	0.495	0.73 (0.16–3.32)	0.681	0.28 (0.07–1.20)	0.086
**Residence**						
Rural	1.00 (Reference)	—	1.00 (Reference)	—	1.00 (Reference)	—
Urban	1.81 (0.56–5.86)	0.322	1.18 (0.42–3.26)	0.753	1.03 (0.37–2.88)	0.953
**Socioeconomic Status**						
**Education**						
College or higher	1.00 (Reference)	—	1.00 (Reference)	—	1.00 (Reference)	—
High school or vocational	2.36 (0.67–8.37)	0.184	1.17 (0.39–3.50)	0.78	2.89 (0.97–8.63)	0.058
Junior high school or lower	2.15 (0.49–9.56)	0.312	2.27 (0.71–7.29)	0.168	2.24 (0.61–8.18)	0.221
**Monthly income (yuan)**						
≥ 5,000	1.00 (Reference)	—	1.00 (Reference)	—	1.00 (Reference)	—
3,000–4,999	1.53 (0.47–4.99)	0.483	1.43 (0.47–4.32)	0.531	0.99 (0.34–2.90)	0.983
< 3,000	3.48 (0.76–15.88)	0.108	3.49 (0.91–13.47)	0.069	1.49 (0.45–4.89)	0.511
**Clinical Characteristics**						
**Cancer type**						
Digestive or urinary	1.00 (Reference)	—	1.00 (Reference)	—	1.00 (Reference)	—
Head, neck, or intracranial	0.71 (0.21–2.43)	0.585	0.98 (0.36–2.62)	0.964	0.37 (0.12–1.09)	0.071
Breast, gyn., or reproductive	1.37 (0.42–4.47)	0.601	0.85 (0.32–2.26)	0.75	1.01 (0.41–2.51)	0.974
Respiratory	2.03 (0.73–5.67)	0.177	1.34 (0.49–3.65)	0.565	0.95 (0.37–2.48)	0.923
**Cancer stage**						
Stage I–II	1.00 (Reference)	—	1.00 (Reference)	—	1.00 (Reference)	—
Stage III–IV	1.19 (0.39–3.59)	0.759	2.71 (1.12–6.54)	**0.027**	2.57 (1.10–5.99)	**0.028**
Pain score, per point	1.00 (0.77–1.29)	0.982	1.02 (0.81–1.28)	0.892	0.91 (0.71–1.15)	0.416
KPS, per 10 points	0.80 (0.53–1.23)	0.312	0.76 (0.49–1.20)	0.245	0.55 (0.36–0.84)	**0.006**

Note. *Values are classification-error-adjusted RRRs (95% confidence intervals); P value. All covariates were entered simultaneously. Reference categories were male sex, college or higher education, unmarried, rural residence, monthly income >=5,000 yuan, digestive or urinary cancer, stage I-II disease and low trajectory membership. P values are two-sided and unadjusted for multiple comparisons. AAQ, Acceptance and Action Questionnaire-II; DAS, Death Anxiety Scale; KPS, Karnofsky Performance Status; RRR, relative risk ratio.*

Table S4. Conditional trajectory probabilities

Direction	Given trajectory	Target trajectory	Conditional probability (%)
P(AAQ | DAS)	Low	Low	91.5
P(DAS | AAQ)	Low	Low	48.3
P(AAQ | DAS)	Low	High	8.5
P(DAS | AAQ)	High	Low	10.8
P(AAQ | DAS)	Moderate	Low	81.3
P(DAS | AAQ)	Low	Moderate	30.5
P(AAQ | DAS)	Moderate	High	18.7
P(DAS | AAQ)	High	Moderate	16.9
P(AAQ | DAS)	High	Low	41.4
P(DAS | AAQ)	Low	High	21.3
P(AAQ | DAS)	High	High	58.6
P(DAS | AAQ)	High	High	72.3

Note. P(AAQ | DAS) denotes experiential-avoidance trajectory membership conditional on observed death-anxiety trajectory membership. P(DAS | AAQ) denotes the reverse conditional probability. These probabilities describe longitudinal co-occurrence and do not indicate causal direction.

Table S5. Joint probabilities of death-anxiety and experiential-avoidance trajectory membership

Death-anxiety trajectory	Experiential-avoidance trajectory	Joint probability (%)	Expected n
Low	Low	34.1	90.7
Low	High	3.2	8.4
Moderate	Low	21.5	57.3
Moderate	High	5.0	13.2
High	Low	15.0	40.0
High	High	21.2	56.5

Note. The six cells represent combinations of three death-anxiety trajectories and two experiential-avoidance trajectories. Expected n was calculated by multiplying each model-estimated joint probability by the analytic sample size.
